# Flexible Transparent Heater Fabricated from Spray-Coated In:ZnO/Ag-NWs/In:ZnO Multilayers on Polyimide Foil

**DOI:** 10.3390/nano12030316

**Published:** 2022-01-19

**Authors:** Rachmat Adhi Wibowo, Katharina Rauchenwald, Stefan Edinger, Neha Bansal, Stefan Diebald, Daniel Habenbacher, Theodoros Dimopoulos

**Affiliations:** 1 AIT Austrian Institute of Technology, Center for Energy, Energy Conversion and Hydrogen, Giefinggasse 2, 1210 Vienna, Austria; rachmat.wibowo@ait.ac.at (R.A.W.); k.rauchenwald@gmx.at (K.R.); stefan.edinger@ait.ac.at (S.E.); neha.bansal@ait.ac.at (N.B.); 2 ATT Advanced Thermal Technologies GmbH, Gewerbeparkstraße 77/Top 5, 8143 Dobl-Zwaring, Austria; stefan.diebald@thermaltech.at (S.D.); daniel.habenbacher@thermaltech.at (D.H.)

**Keywords:** spray-pyrolysis, ZnO, silver nanowires, flexible substrates, transparent heaters, transparent conducting materials

## Abstract

A flexible transparent heater is presented, based on an all-sprayed composite architecture of indium-doped zinc oxide (IZO) layers that sandwich a network of silver nanowires, on a polyimide-foil substrate. This architecture could be materialized through the development of a low-temperature (240 °C) spray-pyrolysis process for the IZO layers, which is compatible with the thermal stability of the transparent polyimide substrate and allows for the formation of compact and transparent layers, without precipitates. The IZO layers entirely embed the silver nanowires, offering protection against environmental degradation and decreasing the junction resistance of the nanowire network. The resulting transparent heaters have a high mean transmittance of 0.76 (including the substrate) and sheet resistance of 7.5 Ω/sq. A steady-state temperature of ~130 °C is achieved at an applied bias of 3.5 V, with fast heater response times, with a time constant of ~4 s The heater is mechanically stable, reaching or surpassing 100 °C (at 3.5 V), under tensile, respectively, compressive-bending stress. This work shows that high-performance transparent heaters can be fabricated using all-sprayed oxide/silver-nanowire composite coatings, that are compatible with large-scale and low-cost production.

## 1. Introduction

Transparent conducting materials (TCMs) combine high visible transparency with high-electrical conductivity. They are applied across a vast number of applications, comprising optoelectronic devices (such as light-emitting diodes, thin-film displays and touch-screens), photovoltaic modules, sensors and actuators, low-emissivity and electrochromic window coatings, etc. [1]. Transparent conducting oxides (TCOs), which in their majority are highly doped, n-type semiconductors, have the widest implementation among TCMs [2]. The most established TCOs are the tin-doped indium oxide (ITO), fluorine-doped tin oxide (FTO) and aluminum-doped zinc oxide (AZO). Although TCOs provide a high performance for the targeted applications, they also have significant disadvantages. One is that they are brittle, which compromises their use in applications demanding flexibility, as well as their processing using roll-to-roll approach. Another disadvantage concerning ITO is that indium is scarce and has a high cost. To address these shortcomings, in recent years, materials such as metallic nanowires, carbon nanotubes, metallic nanofilms and meshes, conducting polymers and combinations of these, have opened new perspectives, functionalities and applications for TCMs [1,3,4,5,6,7]. Additionally, advantageous for certain emerging TCMs is their ability to be processed by non-vacuum-based techniques, particularly from solutions, which decreases capital expenditure and maintenance costs for deposition equipment. 

A wide range of the aforementioned TCMs have found application as transparent heaters (THs) [8], which exploit the Joule heating produced when current flows through a conductor of resistance *R*. Implemented materials in THs include TCOs (the mainstream TH material), carbon-based nanomaterials [9,10], silver or copper nanowires (NWs) [11,12,13,14,15,16], conducting polymers [17] and TCO/metal (Ag or Cu)/TCO multilayers [18,19,20]. Precise and rapid temperature control can be achieved through the electrical properties of the employed materials and the heater design characteristics, which also need to be matched to the input bias or current requirements of the particular application. 

The ideal TH should have the following characteristics: (a) low sheet resistance that yields high heating power density and allows for large-area applications, (b) high resistance uniformity to provide uniform temperature distribution during operation and to avoid hotspots that would potentially lead to heater degradation and eventually breakdown, (c) high transparency in the visible, (d) fast heating rate and high attainable temperature, which depend on the thermal properties of both the heating film and the substrate, (e) high environmental, chemical and mechanical stability, (f) low-cost in terms of materials and fabrication, (g) self-limiting maximum temperature to eliminate the need of additional control electronics. 

THs, based on TCOs and nanomaterials, offer homogeneous optical appearance and temperature profile, which make them attractive with respect to traditional, wire-based heating elements. High optical transmittance and visual homogeneity are of high priority for heating applications regarding displays and smart windows. Additionally, the targeted temperature depends on the application. For example, defogging in automotive needs relatively low temperatures, whereas defrosting requires much higher temperatures. Especially in the automotive sector, current revolutionary trends such as autonomous driving, further push the market demand for THs (e.g., for de-/anti-icing sensors of autonomous driving assistance systems). A wide list of application sectors for THs, including wearables, medical devices, gas sensors and others, has been presented in recent reviews [8,21]. To address these fast-growing markets, flexible THs are particularly attractive since they can be 3D-formed to the product’s shape, are lightweight and can be produced at a low cost by high-throughput, non-vacuum processes, including roll-to-roll. However, the temperature stability of most polymer substrates is a challenging problem. 

Ultrasonic spray-pyrolysis (USP) is an industry-relevant deposition technique that has been extensively used to fabricate a wide variety of TCO materials [22], including doped ZnO. Efforts have also been made to produce high-quality TCO layers (in terms of compactness, transparency and conductivity) at lower substrate temperatures, using more sustainable solution recipes, thereby reducing or completely eliminating organic solvents. In recent publications from our group, we reported on highly transparent and conductive In-doped ZnO (IZO) layers by USP from water-based solutions at temperatures as low as 360 °C [23,24]. However, even this reduced substrate temperature is not low enough to permit the use of polymer substrates. Further reduction of the temperature to below 300 °C requires modification of the solution chemistry in order to obtain TCO layers with suitable properties. 

This work reports on a composite flexible TH, based on IZO layers, sandwiching a network of Ag-NWs, with all of the material components spray-coated in ambient atmosphere, using sustainable processes. To achieve this, a low temperature (240 °C) USP process for the IZO layers was developed, using a water-based solution recipe, whereas a transparent polyimide foil was used as a substrate. The flexible composite electrode was characterized in terms of its structural, electrical, optical and heating properties, achieving temperatures of ~130 °C at a relatively low applied bias (3.5 V), as well as fast heating response times. We thus demonstrate high-performance, TCO/Ag-NW composite, flexible and transparent heaters that are fabricated using a low-cost, spray-coating approach. 

## 2. Materials and Methods

Flexible transparent heaters of IZO/Ag-NWs/IZO were deposited on 50 µm-thick, transparent polymide (PI) substrates (Yangzhou Guotai Fiberglass Co., Yangzhou, China), by sequential spray deposition of (a) bottom IZO layer, (b) Ag-NWs and (c) top IZO layer, using a Sono-Tek, ExactaCoat^®^ (Sono-Tek, Milton, NY, USA) coating system, equipped with a 120 kHz Impact^®^ ultrasonic nozzle, in horizontal geometry. The substrates resided on a hot-plate covered with a high-temperature Al_2_O_3_-SiO_2_ glass ceramic (McMaster-CAR, Cleveland, OH, USA), the temperature of which was precisely monitored and automatically regulated. The fine droplet mist of the solutions was directed from the nozzle towards the hot plate in a fan-shaped spray-pattern, obtained by a controlled jet of air. The nozzle-to-substrate distance was 14 cm. 

The PI substrates were firstly cut into 3.0 × 2.5 cm^2^ pieces and subsequently cleaned in isopropanol and de-ionized water for 15 min at each step, followed by drying in nitrogen gas stream. The samples were then fixed on the substrate holder of the spray coater by placing heavy pieces of glass at the shorter sample edges, defining a deposited area of 2.5 × 2.5 cm^2^. For upscaling demonstration, samples with a deposited area of 5 × 5 cm^2^ were also fabricated.

The used solution recipe for the deposition of IZO is based on previous publications from our group [23,24]. In the present case, however, the recipe was modified for deposition at significantly lower temperature, below 300 °C. This is critical to prevent the deformation of the transparent PI substrate, when subjected to high temperatures for prolonged deposition durations. To this end, the zinc acetate precursor used in the previous recipes, was replaced by zinc acetylacetonate, which decomposes at lower temperatures during spray pyrolysis [25]. The water-based precursor solution contained 0.2 M zinc acetylacetoanate dihydrate (Zn(Acac)_2_ × 2H_2_O) and 0.008 M indium acetate (In(Ac)_3_) as Zn and In sources, respectively, along with 1.8 M acetic acid (HAc), as a complexing agent. All chemicals were purchased from Sigma Aldrich, Austria and used without further treatment. The precursor solution pH was 3. 

On the other hand, the Ag-NW layers were spray-deposited from a solution containing commercial Ag-NW dispersion (Agnw-60, ACS Materials LLC, Pasadena, CA, USA) and isopropanol (99.9% purity) in a 1:100 volume ratio (Sigma-Aldrich, Vienna, Austria). According to the manufacturer, the length of the wires is 20–30 µm and their average diameter 60 nm. Besides, polyvinylpyrrolidone (PVP) was used as a stabilizing agent for the Ag-NWs, with <0.5 wt% left in the final product. The Ag-NWs solution was homogenized by shaking for 2 h prior to the spray deposition. 

The spray parameters used for the IZO and Ag-NW layers are shown in Table 1, corresponding to the coatings with optimized structural, electrical and optical properties. A particular spray pattern was applied for the depositions, as it was introduced in reference [24]. For reasons of comparison, samples without the bottom IZO layer were also fabricated, as well as a sample with a single layer of Ag-NWs, deposited at 150 °C. 

The surface morphology of the layers was investigated by scanning electron microscopy (SEM, Carl Zeiss Supra 40, Oberkochen, Germany), with an acceleration voltage of 5 kV and in-lens detector. The SEM is additionally equipped with an EDAX Octane Elect Plus detector (AMETEK, Berwyn, PA, USA) to perform energy dispersive X-ray spectrometry (EDX). For EDX measurements, the acceleration voltage was increased to 15 kV and the APEX software (AMETEK, Berwyn, PA, USA) was used for spectrum analysis and elemental quantification. The crystallographic information of all layers was studied with grazing incidence X-ray diffraction (GIXRD, Equinox 100, Thermo Fischer Scientific, Munich, Germany) at an incidence angle of 2 deg, using Cu Ka X-ray source with *λ* = 1.54 Å. Specular and total optical transmittance spectra were measured using a Fourier transform spectrometer (FT, Vertex 70, Bruker, Ettlingen, Germany) equipped with an integrating sphere and GaP and Si detectors. A four-point-probe tool (Nagy SD-600, Gäufelden, Germany) was employed to measure the sheet resistance of the coatings. 

**Figure 1:**
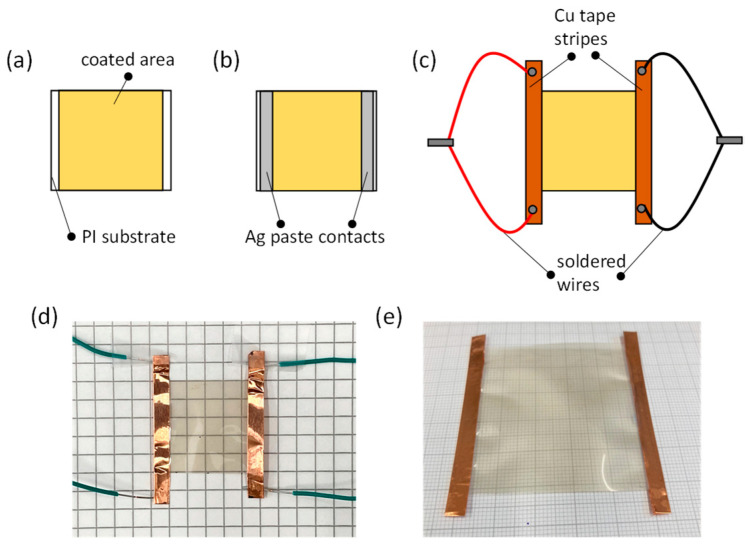
(**a**–**c**) Schematic representation of the fabrication steps of the transparent heater, showing (**a**) the coated area, (**b**) the state after application of the Ag paste and (**c**) after the application of the Cu tape stripe and the soldering of the wires. (**d**) Photo of the transparent heater, featuring the Cu stripes and the soldered wires. (**e**) Photo of a transparent heater with a coated area of 5 × 5 cm^2^.

For the preparation of the transparent heaters, conductive Ag paste (SCP03B, Electrolube, Surrey, UK) was applied along the two opposite edges of the samples (Figure 1a,b) and left for several hours to dry. Afterwards, Cu adhesive tape (1181, 3M Scotch, Vienna, Austria) was applied on the Ag paste and folded to cover the front and the back side of the substrate’s edge (Figure 1c), defining stripes of ~4.5 mm width. The Cu-tape stripes extended beyond the length of the samples. Close to the extremities of the Cu stripes, wires were soldered and the two wires from each stripe were connected, as shown in Figure 1c, at the same terminal of the power supply. For the final heater, the transparent area is 2.2 × 2.5 cm^2^ (see photo in Figure 1d). A photo of a fabricated TH with a coated area of 5 × 5 cm^2^ is also shown in Figure 1e. 

The temperature of the heater was measured with a remote IR sensing device (PeakTech 4960, Ahrensburg, Germany) and monitored continuously. The measurements were realized with the IR beam impeding on the PI foil side. The thermal emissivity of the PI foil was taken as 0.7, according to the literature for transparent polyimides of similar properties [26]. For certain control measurements, the K-type thermocouple of the device was used instead. Bias was applied to the heater through a Keithley 2400 Sourcemeter, measuring the current. Thermography images of the heaters were acquired with a Jenoptic VarioCam HD camera (Jenoptic, Jena, Germany) and processed with the IRBIS 3 analysis software (InfraTec, Dresden, Germany). All measurements were conducted with the heaters free-standing (not in contact with any surface).

## 3. Results and Discussion

The IZO/Ag-NWs/IZO architecture of the transparent heaters offers protection to the Ag-NWs from ambient humidity and other environmental influences. The bottom IZO layer is employed for multiple reasons, namely, (a) it can act as a diffusion barrier between the substrate and the Ag-NWs during the TH deposition and operation, (b) it improves the substrate wettability in the case where techniques such as rod-coating, spin-coating or doctor-blading are used for the Ag-NWs deposition, (c) it improves the NW junction resistance, as it has reproducibly led to lower sheet resistance values, as compared to the case where only the top IZO layer was used. It has been also shown in various works in the literature that the coating of Ag or Cu NW networks with oxide layers (deposited by atomic layer deposition in vacuum or atmospheric pressure) has multiple beneficial effects. These benefits include the improvement of the mechanical stability and the homogeneity of the current density throughout the electrode, which consequently improves the temperature distribution and the onset of the electrical failure [15,16,27,28]. These are therefore strong motivations for the pursued multicomponent architecture. 

**Figure 2:**
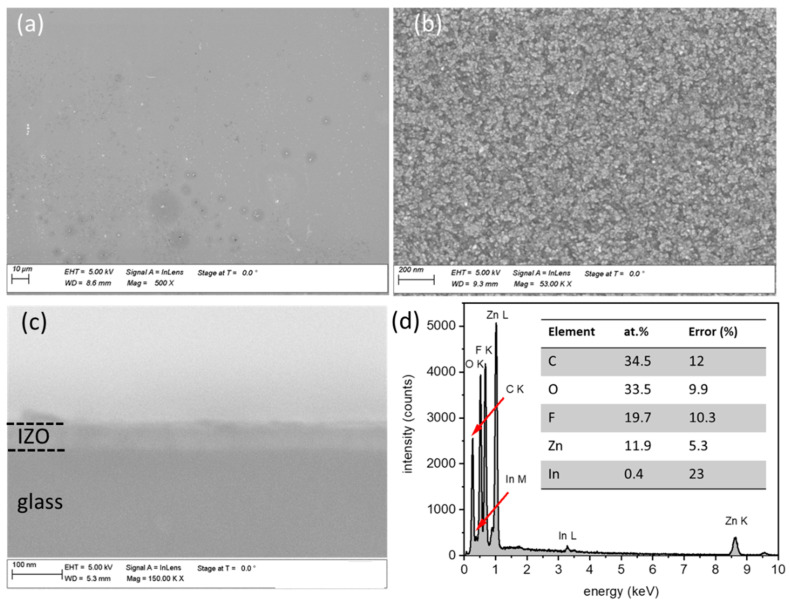
The morphology of the IZO layer on the PI substrate, as observed in (**a**) low magnification and (**b**) high magnification SEM images. (**c**) Cross section SEM image of an IZO layer deposited on glass (simultaneously with the one on PI substrate). (**d**) EDX spectrum with the elemental composition for the IZO film on the PI substrate.

The sprayed IZO layer on the PI substrate shows a dense and compact surface morphology, with small nanocrystals distributed uniformly over the substrate, as seen in the low and high magnification images of Figure 2a,b, respectively. The nanocrystalline nature of the IZO layer is a consequence of its deposition at the reduced temperature of 240 °C, which is the lowest reported in the literature. Depositions at higher substrate temperature (>260 °C) led to the plastic deformation and damaging of the ultra-thin and transparent PI substrate, whereas pyrolysis at lower substrate temperature than 240 °C gives rise to precipitates on the substrate, due to the incomplete decomposition of the precursors and/or incomplete evaporation of the solvent before reaching the substrate. The thickness of the IZO layer is ~50 nm, extracted from SEM cross section image for an IZO layer deposited on glass substrate, as shown in Figure 2c. The IZO layer exhibits a high sheet resistance, in the range of MΩ/sq. The contribution therefore of the IZO layer to the electrical conductivity of the whole electrode can be considered negligible, as the latter is determined by the conductivity of the Ag-NW network. However, the doping of the ZnO with In is important, as In doping leads to the observed compact morphology of the layer. Spray-pyrolysed, un-doped ZnO layers generally form platelets and other nano-structures that would not fit the purpose of the targeted devices [23]. The composition of the IZO layer on the PI was measured by EDX. The EDX spectrum, together with the elemental compositions table are shown in Figure 2d. The In/(In + Zn) concentration in the film is extracted to be 3.4 at.%, close to the molar concentration of the In precursor in the solution.

**Figure 3:**
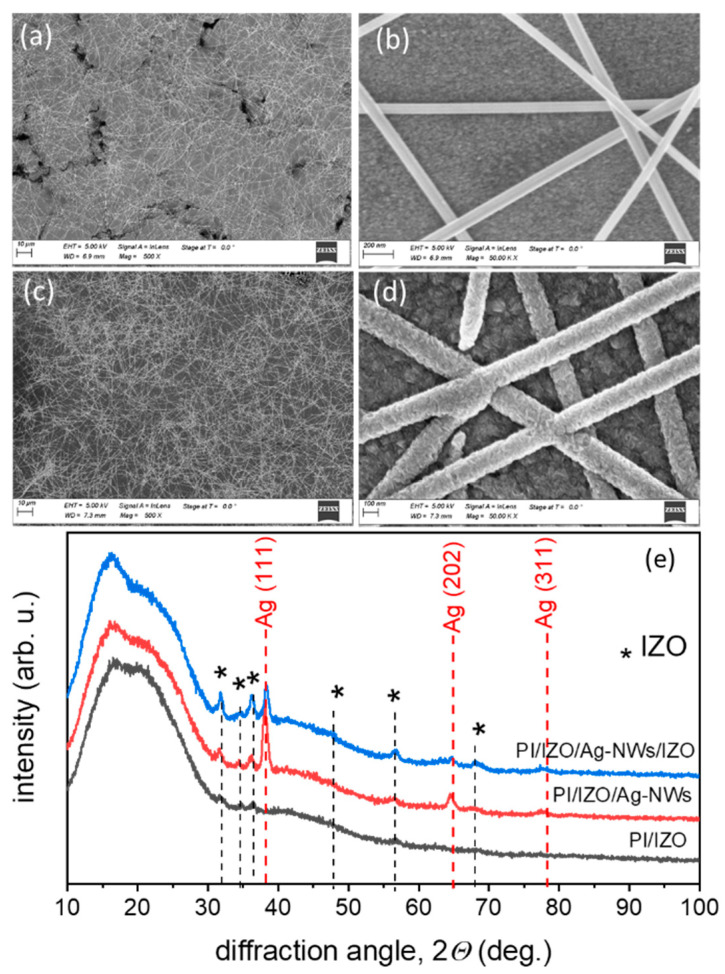
SEM images of PI/IZO/Ag-NWs at (**a**) low magnification and (**b**) high magnification. SEM images of the PI/IZO/Ag-NWs/IZO multilayer at (**c**) low magnification and (**d**) high magnification. (**e**) GIXRD of the PI/IZO, PI/IZO/Ag-NWs and PI/IZO/Ag-NWs/IZO. With asterisk (*) are marked the IZO wurtzite reflections of (100), (002), (101), (012), (110) and (112) from low to higher diffraction angle, according to Crystallography Open Database (COD) 96-101-1259 reference.

The deposition of the Ag-NWs on the PI/IZO yields uniformly distributed Ag-NWs, without agglomerations, as shown in Figure 3a. A network of spray-deposited Ag-NWs can be seen along with the exposed IZO layer beneath. In the higher magnification image of Figure 3b single Ag-NWs with a diameter of 50–70 nm can be distinguished (in accordance with the nominal average diameter), together with the nanocrystalline morphology of the bottom IZO layer. The sheet resistance of the Ag-NW layer varies from sample-to-sample (in the range up to several tens Ω/sq.), suggesting that spraying at this substrate temperature does not reproducibly achieve intimate contact between the wires, and that the wire-to-wire junction resistance is significant. 

The spray pyrolysis of the IZO topmost layer on PI/IZO/Ag-NWs also occurs without the appearance of precipitation, as seen in Figure 3c. The high magnification SEM image (Figure 3d) shows that the wires are thoroughly and conformally covered with IZO, increasing their diameter to 150–200 nm. The thickness of the topmost IZO layer is estimated to be ~60 nm. The complete PI/IZO/Ag-NWs/IZO multilayer has a sheet resistance of 7.5 ± 0.5 Ω/sq., with the error representing the deviation from sample-to-sample. This suggests that intimate contact and reduction of the junction resistance between the Ag-NWs were achieved during the high temperature pyrolysis of IZO, without oxidation of the wires themselves that could be caused by the relatively high processing temperature. It was therefore concluded that, with the first spraying scans of the topmost IZO layer, the oxidation of the Ag-NWs can be prevented. Besides, it was shown that samples employing only the top IZO layer had a higher sheet resistance of 9 ± 1 Ω/sq. This is attributed to a less efficient mitigation of charge carriers at the vicinity of Ag-NW junctions, as compared to the case where two IZO layers sandwich the NW network. Finally, the sample with larger deposited area of 5 × 5 cm^2^ (Figure 1e) showed very similar sheet resistance (~7 Ω/sq.) after the required adjustment of the spraying area has taken place, showing the up-scalability of the spraying process. 

The GIXRD pattern (angle of incidence 2°) of the IZO layer on PI is depicted in Figure 3e. Although the peaks are of low intensity (due to the small thickness of the layer), the layer crystallizes in the typical wurtzite hexagonal ZnO structure, as a complete set of corresponding reflection peaks can be identified (COD 96-101-1259). Nevertheless, no particular texture for this ultra-thin film prevails. After the deposition of the Ag-NWs, new Ag (111), (202) and (311) reflections (COD 96-151-2488) in addition to the wurtzite ZnO ones, appear. The subsequent spray pyrolysis of the IZO topmost layer enhances the wurtzite reflections intensity, as the overall oxide thickness is increased. The presence of the IZO topmost layer also reduces the Ag-NWs reflection intensity as the Ag-NWs are coated by IZO. 

**Figure 4:**
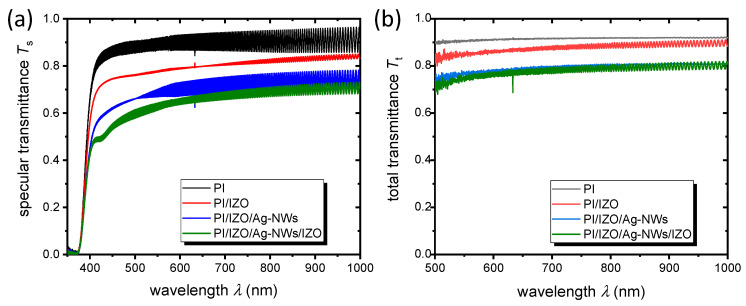
Specular (**a**) and total transmittance (**b**) spectra of PI, PI/IZO, PI/IZO/Ag-NWs and PI/IZO/Ag-NWs/IZO (multi)layers.

The optical property of the multilayers was evaluated using specular *T*_s_ and total *T*_t_ transmittance spectra at the wavelength range of 350–1000 nm and 500–1000 nm, respectively (Figure 4a,b). The high frequency oscillations seen in the spectra are due to light interferences caused by the ultra-thin PI substrate. Table 2 shows values of the mean specular transmittance in the whole visible range 380–750 nm, as well as in the range 500–750 nm. Additionally, the mean total transmittance in the 500–750 nm range along with the diffuse transmittance (*T*_d_ = *T*_t_ − *T*_s_) in the same range is illustrated. For the entire electrode, the total transmittance is 0.76, while the diffuse part amounts to 0.11 (Table 2). Taking into account the total transmittance (0.76) and the sheet resistance (7.5 Ω/sq.) of the electrode, one can calculate the Haacke figure of merit [29], using the relation: FoM = *T*_t_^10^/*R*_s_, and extract FoM = 8.6 × 10^−3^ Ω^−1^. This value is at least as high as for the electrodes in the literature employing transparent, 50 µm-thick PI substrate and a similar architecture, namely either AZO/Ag-NWs/AZO or GZO/Ag-NWs/GZO (GZO: Ga-doped ZnO), with sputtered AZO and GZO layers [30,31]. The FoM is 23.0 × 10^−3^ Ω^−1^ if we consider the total transmittance relative to the substrate (for reasons of comparison with other publications), which is 0.84 (substrate transmittance is 0.91). This value is, indeed, within the range for similar electrodes in the literature employing Ag-NWs coated with different oxide layers, on glass substrates [15,32]. 

**Figure 5:**
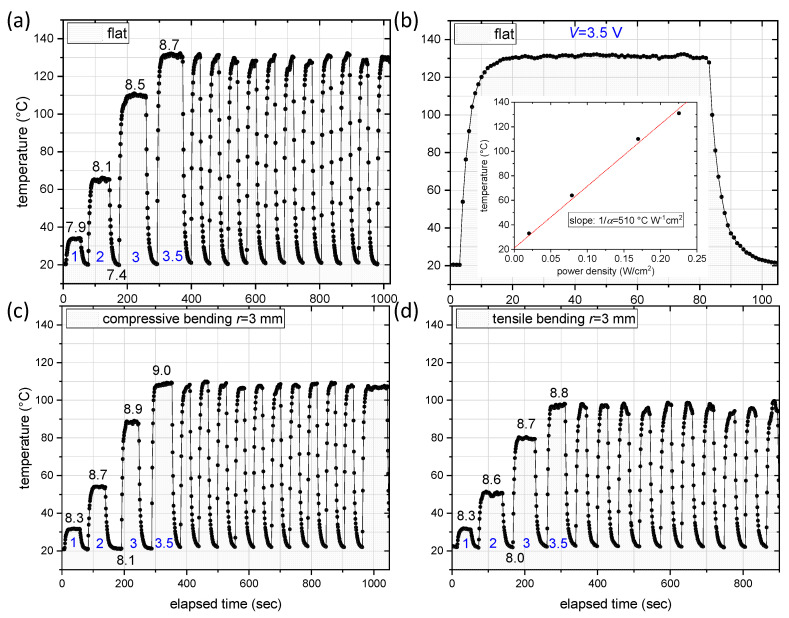
(**a**) Measured temperature versus time for flat heater, upon application of different bias (1, 2, 3 and 3.5 V), followed by ON/OFF cycles, with 3.5 V applied during the ON state. The bias (in V) is marked in blue, whereas the resistance measured at the steady-state temperature for each applied bias, as well as the resistance at RT, are marked in black (in Ω). (**b**) Zoom-in a temperature cycle, including the ramp-up, plateau and ramp-down phases, for the flat heater case. The inset shows the steady-state temperature for each bias, as a function of the power density. (**c**) Measured temperature versus time, following the same measurement protocol as before, for a heater subjected to compressive bending, with a bending radius of 3 mm. (**d**) Measured temperature versus time, following the same measurement protocol as before, for a heater subjected to tensile bending, with a bending radius of 3 mm.

**Figure 6:**
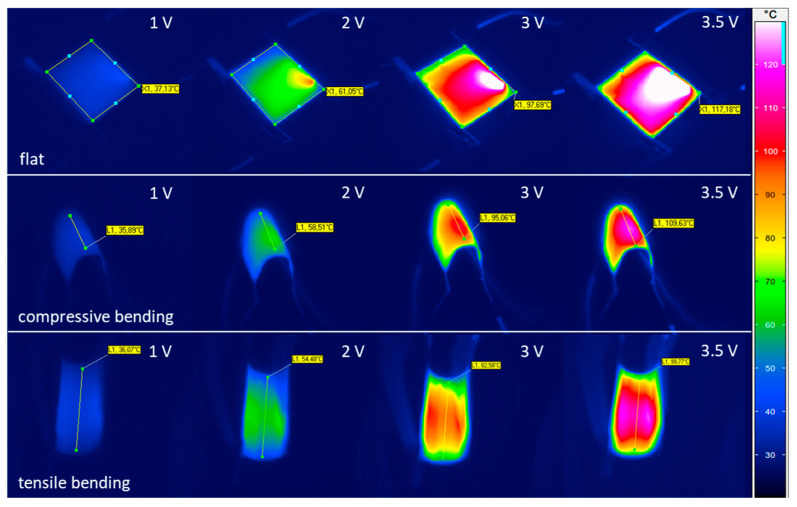
Thermal images of the heater at its flat and bended states (compressive and tensile), for different applied voltages. Additionally, shown are mean temperatures withing the marked areas or lines of the heaters.

As the next step, the electrodes were tested with respect to their heating performance, firstly with the heater at a flat state (Figure 5a). The measured resistance from one contact (Cu stripe) of the sample to the other, at room temperature (RT = 21 °C), was 7.4 Ω (Figure 6). The applied bias between the contacts was progressively set to V = 1, 2, 3 and finally at 3.5 V. At each bias, the temperature of the heater was measured until it reached a plateau, i.e., the steady-state temperature, $T_{st}$, was reached. After reaching the plateau, the bias was turned off and the sample was left to cool down. For the bias of V = 3.5 V, ON/OFF cycles of the bias were also realized, with each cycle’s duration being 1 min (30 s ON and 30 se OFF). For the flat electrode, the obtained $T_{st}$ at each bias were: 34 °C (1 V), 65 °C (2 V), 110 °C (3 V) and 131 °C (3.5 V). The resistance of the heater increased with the temperature, from 7.4 Ω at 21 °C to 8.7 Ω at ~130 °C, as expected from the metallic nature of the Ag-NWs. During the ON/OFF cycles, the maximum attained temperature was reproducibly around 130 °C. Figure 5b shows a zoom-in of the ramp-up phase, the plateau and the ramp-down phase of the heater, for V = 3.5 V. The ramp-up time from RT to the steady-state temperature was ~15 s. 

If we consider relatively small temperature variations (neglecting non-linear radiation losses) and assuming that the heat capacity of the substrate is much larger than the one of the coating, the following formula can be written, for the time evolution of the heater temperature [8,13]:

(1) $$T\left(t\right)\approx T_{0}+\frac{1}{\alpha }\frac{P}{A}\left[1-exp\left(-\frac{t}{\tau }\right)\right],\;with\;\tau =\frac{\rho_{sub}t_{sub}c_{sub}}{\alpha }$$

τ is the time constant of the temperature rise (corresponding to a $\left(T\left(t\right)-T_{0}\right)$ that is 63% of the $T_{st}$), which depends on the density, $\rho_{sub}$, thickness, $t_{sub}$, and specific heat capacity, $c_{sub}$, of the substrate, while α is the heat transfer constant. $P=V^{2}/R$ is the joule-heating power, A the area of the heater (2.5 × 2.5 cm^2^) and $T_{0}$ the initial temperature. The steady-state temperature can be therefore written as:

(2) $$T_{st}\approx T_{0}+\frac{1}{\alpha }\frac{P}{A}$$

By plotting the $T_{st}$ as a function of the power density P/A (inset of Figure 5b) and by linear fitting of the data according to Equation (2) (for $\;T_{0}=21$ °C), we extract the heat transfer constant α = 19.6 × 10^−3^ W/(°C cm^2^) from the slope of the fit (slope = 1/α). The slope, representing the thermal resistance, is 510 °C cm^2^/W. For the PI substrate: $\;t_{sub}=50$ µm, $\;\rho_{sub}=1.40$ gr/cm^3^ and $\;c_{sub}=1.09$ J/(gr °C). Substituting these values in Equation (1), we extract the time constant to be: τ ≈ 4 s. This agrees with the time constant extracted from the temperature ramp-up shown in Figure 5b. The short time constant is due to the low thickness of the used PI substrate and is similar to results in the literature using similar substrate [31,33]. 

When compressive bending is applied to the TH, with a bending radius of *r* = 3 mm (Figure 5c), the attained measured temperatures are lower compared to the flat heater case, whereas the resistance is somewhat increased (by 0.7 Ω at RT). Specifically, the temperature values were: 32 °C (1 V), 54 °C (2 V), 88 °C (3 V) and 109 °C (3.5 V). In the case of tensile bending, with the same bending radius of *r* = 3 mm (Figure 5d), the measured attained temperatures were further reduced to: 32 °C (1 V), 50 °C (2 V), 80 °C (3 V) and 98 °C (3.5 V). For both compressive and tensile bending, the ON/OFF cycling shows that the maximum temperature can be reproducibly attained, with fast ramp-up times.

The temperature of the flat heater, residing on a plexiglass support, was also measured with the K-type thermocouple of the instrument residing on the heater’s surface, verifying that the contactless IR measurement agrees with the steady-state temperature measured using the thermocouple. The investigated heaters are also highly stable. After storing the heater for 7 weeks in ambient conditions, the temperature profile was again measured at an applied bias of 3.5 V (flat heater). The heater resistance (from one contact to the other) was measured 8.3 Ω at RT, somewhat higher than the value in Figure 5a. The steady-state temperature attained was 126 °C, with the resistance at this temperature being 9.4 Ω. As the voltage was ramped gradually up, electric breakdown of the heater took place at 6 V, with the temperature reaching ~220 °C before the breakdown and the resistance approaching 12 Ω. After the breakdown, a crack that run from one side of the substrate to the other, along the contact, appeared, which shows that the breakdown was caused by PI degradation induced by the high temperature, which can locally exceed the substrate’s stability threshold. 

At this point, for reasons of comparison, it is noteworthy to refer to the sample featuring one single layer of Ag-NWs, deposited at a higher temperature (150 °C), in order to decrease its sheet resistance to 15 Ω/sq. When subjected to the same heating procedure, the heater reached ~130 °C at a voltage of 7 V (corresponding to 0.37 W/cm^2^). Under these conditions electrical break-down took place, which marks a significantly lower performance with respect to the embedded NWs in IZO.

The obtained thermal images of the embedded Ag-NWs heaters at their flat and bended states, are shown in Figure 6. Additionally, average temperature values over the marked areas for the flat heaters and marked lines for the bended ones are shown. It needs to be pointed out that, due to the very small thickness of the foil, ripples on its surface cannot be avoided, especially at the proximity of the contacts where pressure had to be applied. These ripples eventually lead to unwanted reflections, influencing the apparent temperature homogeneity. For the bended samples, reflections are more pronounced. 

At this point, a comparison can be made to other THs from the literature combining Ag-NWs with transparent polyimide. Huang et al. [34] reported on embedded Ag-NWs in 50 µm-thick transparent PI, with sheet resistance of 5.6 Ω/sq. and transmittance of 0.58 at 550 nm. The reported thermal resistance of the TH (size of 2.5 × 2.5 cm^2^) was ~161 °C cm^2^/W, significantly lower than the one obtained in the present work (510 °C cm^2^/W), showing the more efficient transduction of electrical energy into joule heating for the IZO/Ag-NWs/IZO THs. Another study by Pyo and Kim [35] reported on embedded Ag-NWs in PI, featuring a relatively high sheet resistance of 23 Ω/sq. and reaching temperatures of up to 160 °C at relatively high bias of 16 V, but without stating the corresponding power density. Furthermore, in a highly relevant study, Wang et al. [31] reported THs on 50 µm-thick PI, based on the GZO/Ag-NWs/GZO architecture, with the GZO layers deposited by sputtering. The reported sheet resistance was 14.6 Ω/sq., with a transmittance similar to the present work (0.79 at 550 nm). The THs (with an area of 2 × 2 cm^2^) reached ~130 °C for a power of ~2.7 W, corresponding to a power density of ~0.68 W/cm^2^, which is three times larger than the value achieved here (~0.23 W/cm^2^). The difference can be at least partially attributed to the lower sheet resistance of the IZO/Ag-NWs/IZO electrode, which is achieved through an intimate NW contact. Finally, the performance of the THs presented here is similar to the one achieved by Gupta et al. [36] for sprayed crack templates followed by Ag evaporation in the crack regions, on a PET substrate (area of 4 × 3 cm^2^), which yielded thermal resistance values as high as 515 °C cm^2^/W. 

## 4. Conclusions

In conclusion, we have presented a flexible transparent heater based on all-sprayed IZO/Ag-NWs/IZO multilayer on polyimide foil. The fabrication was possible through the development of a low-temperature (240 °C) spray-pyrolysis process for the IZO layers, which is compatible with the thermal stability of the transparent polyimide. The nanocrystalline IZO layers are compact and highly transparent, entirely embedding the Ag-NWs. This offers environmental protection and decreases the junction resistance of the Ag-NW network, leading to reproducibly low sheet resistance. The fabricated transparent heaters have a high mean transmittance of 0.76 (including the substrate) and sheet resistance of 7.5 Ω/sq. As a result of this optimized architecture, a steady-state temperature of ~130 °C was achieved at an applied bias of 3.5 V, with fast temperature response times of several seconds. The heater is mechanically stable, with attained temperatures reaching or surpassing 100 °C (at 3.5 V), under tensile, respectively, compressive bending stress. This work shows that high-performance transparent heaters can be fabricated using all-sprayed oxide/silver-nanowire composite coatings, compatible with large-scale and low-cost production.

## Figures and Tables

**Table 1:** Spray parameters for depositing IZO/Ag-NWs/IZO on PI substrates.

Spray Sequence Number	Material	Substrate Temperature (°C)	Flow Rate (mL/min)	Shaping Air Overpressure (bar)	Scan Cycles
1	IZO	240	0.1	0.2	500
2	Ag-NWs	100	0.4	9.0	3
3	IZO	240	0.1	0.2	600

**Table 2:** Mean transmittance values for two wavelength ranges.

Sample	*T*_s_ (380–750 nm)	*T*_s_ (500–750 nm)	*T*_t_ (500–750 nm)	*T*_d_ = *T*_t_ − *T*_s_ (500–750 nm)
PI	0.86	0.90	0.91	0.01
PI/IZO	0.75	0.79	0.86	0.07
PI/IZO/Ag-NWs	0.65	0.70	0.80	0.10
PI/IZO/Ag-NWs/IZO	0.60	0.65	0.76	0.11

## Data Availability

Data presented in this paper could be made available from the corresponding author upon request.

## References

[1] Levy D., Castellón E. (2018). Transparent Conductive Materials: Materials, Synthesis, Characterization, Applications.

[2] Ellmer K. (2012). Past Achievements and Future Challenges in the Development of Optically Transparent Electrodes. Nat. Photonics.

[3] Qin L.-H., Yan Y.-Q., Yu G., Zhang Z.-Y., Zhama T., Sun H. (2021). Research Progress of Transparent Electrode Materials with Sandwich Structure. Materials.

[4] Zhang Y., Ng S.-W., Lu X., Zheng Z. (2020). Solution-Processed Transparent Electrodes for Emerging Thin-Film Solar Cells. Chem. Rev..

[5] Hofmann A.I., Cloutet E., Hadziioannou G. (2018). Materials for Transparent Electrodes: From Metal Oxides to Organic Alternatives. Adv. Electron. Mater..

[6] Zilberberg K., Riedl T. (2016). Metal-Nanostructures—A Modern and Powerful Platform to Create Transparent Electrodes for Thin-Film Photovoltaics. J. Mater. Chem. A.

[7] Sannicolo T., Lagrange M., Cabos A., Celle C., Simonato J.-P., Bellet D. (2016). Metallic Nanowire-Based Transparent Electrodes for Next Generation Flexible Devices: A Review. Small.

[8] Papanastasiou D.T., Schultheiss A., Muñoz-Rojas D., Celle C., Carella A., Simonato J., Bellet D. (2020). Transparent Heaters: A Review. Adv. Funct. Mater..

[9] Yoon Y.-H., Song J.-W., Kim D., Kim J., Park J.-K., Oh S.-K., Han C.-S. (2007). Transparent Film Heater Using Single-Walled Carbon Nanotubes. Adv. Mater..

[10] Du J., Pei S., Ma L., Cheng H.-M. (2014). 25th Anniversary Article: Carbon Nanotube- and Graphene-Based Transparent Conductive Films for Optoelectronic Devices. Adv. Mater..

[11] Celle C., Mayousse C., Moreau E., Basti H., Carella A., Simonato J.-P. (2012). Highly Flexible Transparent Film Heaters Based on Random Networks of Silver Nanowires. Nano Res..

[12] Kim T., Kim Y.W., Lee H.S., Kim H., Yang W.S., Suh K.S. (2013). Uniformly Interconnected Silver-Nanowire Networks for Transparent Film Heaters. Adv. Funct. Mater..

[13] Sorel S., Bellet D., Coleman J.N. (2014). Relationship between Material Properties and Transparent Heater Performance for Both Bulk-like and Percolative Nanostructured Networks. ACS Nano.

[14] Ergun O., Coskun S., Yusufoglu Y., Unalan H.E. (2016). High-Performance, Bare Silver Nanowire Network Transparent Heaters. Nanotechnology.

[15] Khan A., Nguyen V.H., Muñoz-Rojas D., Aghazadehchors S., Jiménez C., Nguyen N.D., Bellet D. (2018). Stability Enhancement of Silver Nanowire Networks with Conformal ZnO Coatings Deposited by Atmospheric Pressure Spatial Atomic Layer Deposition. ACS Appl. Mater. Interfaces.

[16] Celle C., Cabos A., Fontecave T., Laguitton B., Benayad A., Guettaz L., Pélissier N., Nguyen V.H., Bellet D., Muñoz-Rojas D. (2018). Oxidation of Copper Nanowire Based Transparent Electrodes in Ambient Conditions and Their Stabilization by Encapsulation: Application to Transparent Film Heaters. Nanotechnology.

[17] Gueye M.N., Carella A., Demadrille R., Simonato J.-P. (2017). All-Polymeric Flexible Transparent Heaters. ACS Appl. Mater. Interfaces.

[18] Lee S.Y., Hwang J.Y. (2020). Transparent Heater with Meshed Amorphous Oxide/Metal/Amorphous Oxide for Electric Vehicle Applications. Sci. Rep..

[19] Ko E.-H., Kim H.-J., Lee S.-J., Lee J.-H., Kim H.-K. (2016). Nano-Sized Ag Inserted into ITO Films Prepared by Continuous Roll-to-Roll Sputtering for High-Performance, Flexible, Transparent Film Heaters. RSC Adv..

[20] Park S.-H., Lee S.-M., Ko E.-H., Kim T.-H., Nah Y.-C., Lee S.-J., Lee J.H., Kim H.-K. (2016). Roll-to-Roll Sputtered ITO/Cu/ITO Multilayer Electrode for Flexible, Transparent Thin Film Heaters and Electrochromic Applications. Sci. Rep..

[21] Gupta R., Rao K.D.M., Kiruthika S., Kulkarni G.U. (2016). Visibly Transparent Heaters. ACS Appl. Mater. Interfaces.

[22] Malik O., Hidalga-Wade F.J.D.L., Amador R.R., Samer M. (2017). Spray Pyrolysis Processing for Optoelectronic Applications. Pyrolysis.

[23] Edinger S., Bansal N., Bauch M., Wibowo R., Újvári G., Hamid R., Trimmel G., Dimopoulos T. (2017). Highly Transparent and Conductive Indium-Doped Zinc Oxide Films Deposited at Low Substrate Temperature by Spray Pyrolysis from Water-Based Solutions. J. Mater. Sci..

[24] Winkler N., Wibowo A., Kubicek B., Kautek W., Ligorio G., List-Kratochvil E., Dimopoulos T. (2019). Rapid Processing of In-Doped ZnO by Spray Pyrolysis from Environment-Friendly Precursor Solutions. Coatings.

[25] Arii T., Kishi A. (2006). Humidity Controlled Thermal Analysis: The Effect of Humidity on Thermal Decomposition of Zinc Acetylacetonate Monohydrate. J. Therm. Anal. Calorim..

[26] Xiao T., Fan X., Fan D., Li Q. (2017). High Thermal Conductivity and Low Absorptivity/ Emissivity Properties of Transparent Fluorinated Polyimide Films. Polym. Bull..

[27] Papanastasiou D.T., Charvin N., Resende J., Nguyen V.H., Sekkat A., Muñoz-Rojas D., Jiménez C., Flandin L., Bellet D. (2021). Effects of Non-Homogeneity and Oxide Coating on Silver Nanowire Networks under Electrical Stress: Comparison between Experiment and Modeling. Nanotechnology.

[28] Nguyen V.H., Resende J., Papanastasiou D.T., Fontanals N., Jiménez C., Muñoz-Rojas D., Bellet D. (2019). Low-Cost Fabrication of Flexible Transparent Electrodes Based on Al Doped ZnO and Silver Nanowire Nanocomposites: Impact of the Network Density. Nanoscale.

[29] Haacke G. (1976). New Figure of Merit for Transparent Conductors. J. Appl. Phys..

[30] Huang Q., Shen W., Fang X., Chen G., Yang Y., Huang J., Tan R., Song W. (2015). Highly Thermostable, Flexible, Transparent, and Conductive Films on Polyimide Substrate with an AZO/AgNW/AZO Structure. ACS Appl. Mater. Interfaces.

[31] Wang R., Cai P., Xu W., Tan R., Shen W., Wang Z., Chen G., Huang J., Fang X., Song W. (2020). Highly Flexible and Transparent Film Heaters Based on Colorless Polyimide Substrate with a GZO/AgNW/GZO Sandwich Structure. J. Mater. Sci. Mater. Electron..

[32] Lagrange M., Sannicolo T., Muñoz-Rojas D., Lohan B.G., Khan A., Anikin M., Jiménez C., Bruckert F., Bréchet Y., Bellet D. (2017). Understanding the Mechanisms Leading to Failure in Metallic Nanowire-Based Transparent Heaters, and Solution for Stability Enhancement. Nanotechnology.

[33] Sui D., Huang Y., Huang L., Liang J., Ma Y., Chen Y. (2011). Flexible and Transparent Electrothermal Film Heaters Based on Graphene Materials. Small.

[34] Huang Q., Shen W., Fang X., Chen G., Guo J., Xu W., Tan R., Song W. (2015). Highly Flexible and Transparent Film Heaters Based on Polyimide Films Embedded with Silver Nanowires. RSC Adv..

[35] Pyo K., Kim J.-W. (2016). Thermally Stable and Flexible Transparent Heaters Based on Silver Nanowire-Colorless Polyimide Composite Electrode. Curr. Appl. Phys..

[36] Gupta R., Rao K.D.M., Srivastava K., Kumar A., Kiruthika S., Kulkarni G.U. (2014). Spray Coating of Crack Templates for the Fabrication of Transparent Conductors and Heaters on Flat and Curved Surfaces. ACS Appl. Mater. Interfaces.

